# Targeting Multidrug-Recalcitrant Pseudomonas aeruginosa Biofilms: Combined-Enzyme Treatment Enhances Antibiotic Efficacy

**DOI:** 10.1128/aac.01358-22

**Published:** 2023-01-05

**Authors:** Yixin Zhang, Wei Wei, Huamei Wen, Zhongle Cheng, Zhongwen Mi, Jing Zhang, Xiaolong Liu, Xinjiong Fan

**Affiliations:** a School of Basic Medical Sciences, Anhui Medical University, Hefei, Anhui, China; b The First Affiliated Hospital of Anhui Medical University, Hefei, Anhui, China; c University of Science and Technology of China, Hefei, Anhui, China; d Stomatological Hospital and College, Key Laboratory of Oral Diseases Research of Anhui Province, Anhui Medical University, Hefei, Anhui, China

**Keywords:** biofilm, quorum sensing, *Pseudomonas aeruginosa*, rational design, *N*-acylhomoserine lactonase

## Abstract

Pseudomonas aeruginosa is an opportunistic pathogen that forms biofilms during infection, resulting in recalcitrance to antibiotic treatment. Biofilm inhibition is a promising research direction for the treatment of biofilm-associated infections. Here, a combined-enzyme biofilm-targeted strategy was put forward for the first time to simultaneously prevent biofilm formation and break down preformed biofilms. The *N*-acylhomoserine lactonase AidH was used as a quorum-sensing inhibitor and was modified to enhance the inhibitory effect on biofilms by rational design. Mutant AidH_A147G_ exerted maximum activity at the human body temperature and pH and could reduce the expression of virulence factors as well as biofilm-related genes of P. aeruginosa. Subsequently, the P. aeruginosa self-produced glycosyl hydrolase PslG joined with AidH_A147G_ to disrupt biofilms. Interestingly, under the combined-enzyme intervention for P. aeruginosa wild-type strain PAO1 and clinical strains, no biofilm was observed on the bottom of NEST glass-bottom cell culture dishes. The combination strategy also helped multidrug-resistant clinical strains change from resistant to intermediate or sensitive to many antibiotics commonly used in clinical practice. These results demonstrated that the combined-enzyme approach for inhibiting biofilms is a potential clinical treatment for P. aeruginosa infection.

## INTRODUCTION

Pseudomonas aeruginosa is an increasingly prevalent opportunistic human pathogen causing a wide range of acute and chronic infections in patients with severe burns, metabolic diseases, and cystic fibrosis. The P. aeruginosa biofilm has strong resistance to antibiotics (1, 2). Along with biofilm formation, the mortality from P. aeruginosa infection increases with the production of various virulence factors such as lectin, exotoxin A, pyocyanin, elastase, and alginate, which assist P. aeruginosa in evading the host’s immune response and causing pathological damage (3, 4). Antibiotics are the main treatment for P. aeruginosa infections (5, 6). However, the emergence and spread of antibiotic-resistant bacteria and the increase in chronic, difficult-to-eradicate infections have led researchers to seek alternative treatments (7, 8). Clinical therapeutic options have thus focused on prevention via the prophylactic use of antibiotics, reductions in the use of implanted devices, the mechanical removal of infection sources, and the optimization of antibiotic regimens (9–11). Few new therapeutic options are available to treat stubborn biofilms that have already formed (12, 13).

Biofilm inhibition is a promising research direction to treat biofilm-associated infections (14). At present, most biofilm-targeted strategies focus on two targets, (i) preventing the formation of biofilms, such as by jamming communication through the inhibition of quorum sensing (QS) (15, 16), and (ii) dispersing existing biofilms (17). For prevention, QS is a key component of biofilm communication that can be used to inhibit developmental processes such as initial attachment, microcolony formation, and maturity (18). In P. aeruginosa, two QS systems (LasI and Rhl) are the most widely studied. They are based on the acylhomoserine lactone molecules *N*-(3-oxododecanoyl)-l-homoserine lactone (3-oxo-C_12_-HSL) and *N*-butanoyl-l-homoserine lactone (C_4_-HSL) (19). These signals can be devitalized by *N*-acyl homoserine lactonases (AHL-lactonases) and can regulate genes related to pathogenicity and biofilm formation (20). The extracellular matrix is the main component of biofilm and plays a significant role in maintaining its stability and structure (21, 22). By dispersing components of the extracellular matrix, biofilm bacteria return to a planktonic state and expose themselves to the host immune system, thereby losing some antibiotic resistance (9). Glycosyl hydrolase for dissociating the exopolysaccharides could thus help degrade established biofilms (23).

The targeted regulation of QS and the targeted dispersal of biofilm components can each influence biofilm recalcitrance. However, considering the complexity of clinical treatment, a single-method approach likely cannot achieve good results across the entire biofilm development process. Moreover, most studies focus on wild-type strain PAO1 and ignore clinical pathogenic strains in practical applications. Therefore, we propose combining anti-QS and biofilm dispersal to simultaneously prevent biofilm formation, break down established biofilms, and even measure the effects on clinical strains. AidH is one *N*-acylhomoserine lactonase with a well-described enzyme structure and degradation mechanism (24, 25). A self-produced glycosyl hydrolase, PslG, efficiently breaks up biofilms of P. aeruginosa (23, 26, 27). The goals of this study were 4-fold: (i) to increase the rate of degradation of signaling molecules of AidH using rational design and characterize the enzymatic activity of wild-type and mutant strains; (ii) to determine the dosage and anti-QS effects of AidH_A147G_ on wild-type strain PAO1 and the anti-QS effects on clinical isolates; (iii) to determine the antibiofilm effects of the anti-QS approach, the induced-dispersal approach, and the combined method on wild-type strain PAO1 and clinical isolates; and (iv) to determine whether the three interventions increase the efficacy of antibiotics against different P. aeruginosa strains.

## RESULTS AND DISCUSSION

### Rational design used to enhance AidH activity.

**FIG 1:**
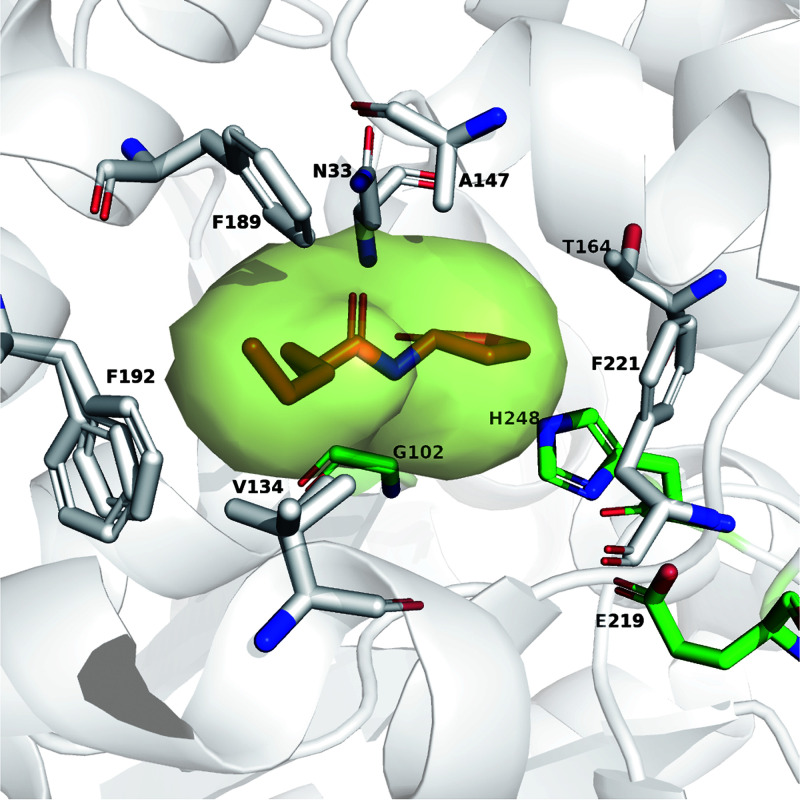
Structural analyses of AidH. The catalytic residues (S102G, E219, and H248) are shown as green sticks, and residues potentially affecting the catalytic activity of the enzymes are shown as white sticks. Prediction of the substrate tunnel was performed by using the CAVER PyMOL 3.0 plug-in.

The crystal structure of the AidH complex with *N*-hexanoyl homoserine lactone was solved (PDB accession no. 4G8B), and residues 5 Å away from the ligand were identified as hot spots (26) (Fig. 1). All proteins were >95% pure by a fast protein liquid chromatography (FPLC) Äkta purifier (see Fig. S1 in the supplemental material). Evaluation of the rates of degradation of C_4_-HSL and 3-oxo-C_12_-HSL by AidH and its mutants indicates that altering the side chain of residue 147 significantly influences the activities of AidH (Table S1). Compared with the wild type, the efficiencies of the hydrolysis of 3-oxo-C_12_-HSL and C_4_-HSL by mutant AidH_A147G_ were increased by 1.4- and 2.2-fold, respectively. In comparison to other lactonases, the *k*_cat_/*K_m_* ratios of AidH (1.59 × 10^5^ s^−1^ M^−1^) and AidH_A147G_ (3.51 × 10^5^ s^−1^ M^−1^) toward C_4_-HSL were higher than those of AiiA (4.38 × 10^3^ s^−1^ M^−1^) (28), SacPox (11.62 s^−1^ M^−1^) (29), and huPON2 (5.4 s^−1^ M^−1^) (30). Besides, the catalytic efficiencies of AidH (1.31 × 10^5^ s^−1^ M^−1^) and AidH_A147G_ (1.86 × 10^5^ s^−1^ M^−1^) toward 3-oxo-C_12_-HSL were higher than those of PvdQLα146W, Fβ24Y (5.8 × 10^3^ s^−1^ M^−1^) (31), GKL (9.3 × 10^3^ s^−1^ M^−1^) (32), and SacPox (2.2 × 10^3^ s^−1^ M^−1^) (29) (Table 1; Fig. S2).

**TABLE 1:** Comparison of kinetic parameters for AidH and the mutants

Substrate and enzyme	Mean *K_m_* (mM) ± SD	Mean *k*_cat_ (s^−1^) ± SD	*k*_cat_/*K_m_* ratio (s^−1^ M^−1^)
C_4_-HSL			
AidH	0.25 ± 0.03	39.80 ± 1.30	1.56 × 10^5^
AidH_A147G_	0.16 ± 0.02	55.13 ± 1.50	3.51 × 10^5^
AidH_A147V_	0.38 ± 0.06	9.79 ± 0.53	2.59 × 10^4^
AidH_A147F_	1.08 ± 0.25	1.42 ± 1.71	1.32 × 10^4^
3-Oxo-C_12_-HSL			
AidH	0.24 ± 0.03	29.45 ± 1.70	1.31 × 10^5^
AidH_A147G_	0.36 ± 0.08	67.20 ± 6.90	1.86 × 10^5^
AidH_A147V_	0.15 ± 0.02	12.58 ± 0.59	8.30 × 10^4^
AidH_A147F_	0.64 ± 0.12	9.30 ± 1.02	1.45 × 10^4^

### AidH and its mutants exert maximum activity under mild reaction conditions, including temperature and pH.

To further characterize AidH and its mutants, the activities under various temperatures and pHs were determined using *p*-nitrophenol acetate as a substrate. For the optimum temperature, we measured relative activities in the range of 25°C to 50°C. AidH and its mutants showed the best activity at 40°C (Fig. S3). In addition, AidH_A147G_ retained approximately 70% of the maximum activity at 35°C and about 60% of the maximum activity at 45°C. Compared to the AHL-lactonases Aii810 (33), AiiK (34), Est816 (35), and AiiA (28), whose optimum temperatures were 20°C, 45°C, 60°C, and 45°C, respectively, AidH possessed better adaptability at human body temperatures.

For the optimum pH, AidH and its mutants all had optimal activity at around pH 7.5 (Fig. S4). AidH_A147G_ retained approximately 70% of the maximum activity at pH 7 and about 80% of the maximum activity at pH 9, showing that AidH has good adaptability to the pH environment in humans. Regarding thermostability, the proteins were incubated at 20°C to 50°C for 24 h. The activities of AidH and the mutants were all reduced slightly at temperatures below 37°C. After incubation at 37°C for 12 h, AidH maintained more than 50% relative activity. After 24 h, it maintained more than 24% relative activity (Fig. S5). However, the thermostability of AidH was slightly lower than that of Aii810 (33), which retained about 60% activity after incubation at 45°C for 24 h. These results show that this enzyme could exert maximum activity under various conditions of the human body.

### Effect of AidH_A147G_ on virulence and biofilm production of P. aeruginosa.

The anti-QS effects of AidH_A147G_ *in vitro* on the production of virulence factors and biofilms of wild-type strains and clinical pathogenic strains were determined. First, wild-type strain PAO1 was used to explore the enzyme dosage. Different concentrations of purified AidH_A147G_ (2 μg/mL to 1 mg/mL) were added to a 12-well cell culture plate. The results showed that AidH_A147G_ played a role in attenuating virulence and biofilm production. It needs to be noted that biofilm inhibition requires more enzyme additions to achieve optimal results, compared to other virulence factors. A dosage of 100 μg/mL AidH_A147G_ was selected for subsequent experiments (Fig. S6).

**FIG 2:**
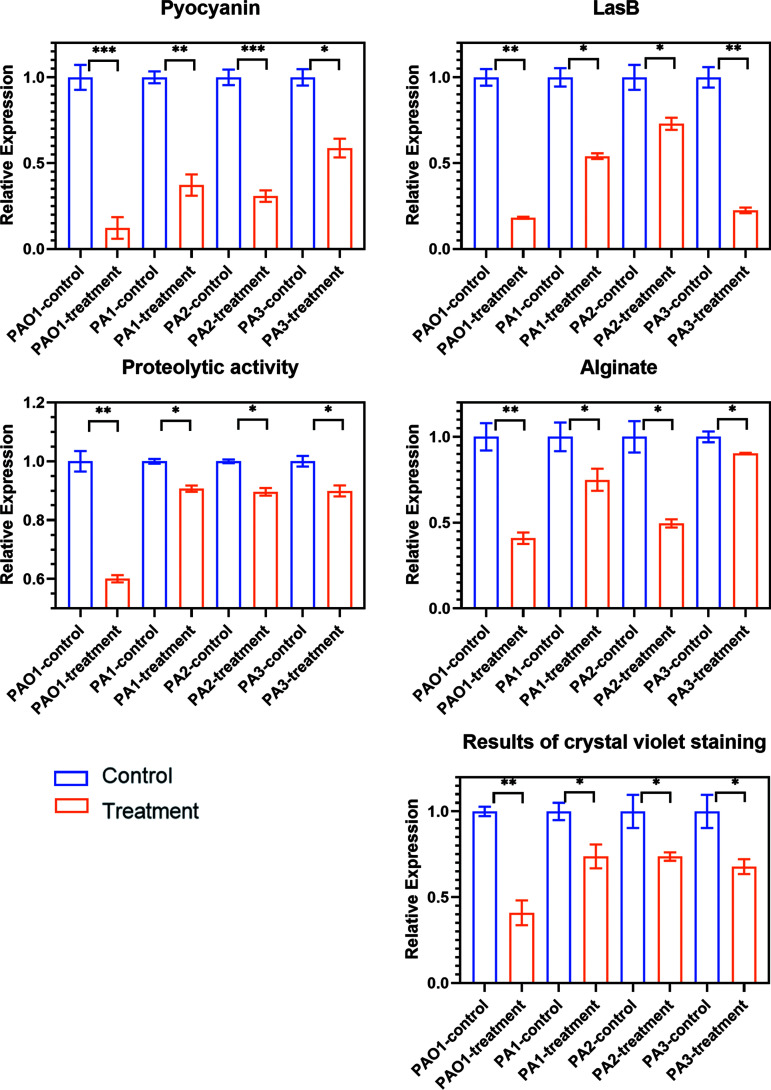
Effect of AidH_A147G_ on the production of virulence factors and biofilms. PAO1 represents P. aeruginosa wild-type strain PAO1, and PA1, PA2, and PA3 represent the clinical isolates. The ordinate represents relative expression. Each data point represents the mean from three replicates. Error bars in each panel represent standard deviations (SD) from triplicates. A *t* test was performed to test differences between groups. *, *P* < 0.05; **, *P* < 0.01; ***, *P* < 0.001.

Compared to the control group, the proteolytic activity of the experimental group decreased by about 40%; LasB elastase decreased by about 80%, pyocyanin decreased by about 90%, and alginate decreased by about 60%. Regarding the effect of biofilm inhibition quantified by crystal violet (CV) staining of AidH_A147G_, the intervention with 100 μg/mL of enzymes reduced PAO1 biofilms by about 60% (Fig. 2). Compared with other AHL-lactonases, AidH_A147G_ had a strong inhibitory effect on some virulence factors and biofilms. AidH_A147G_ had better inhibitory effects on LasB and pyocyanin than did AhlS added at 2,000 μg/mL (36). In a previous study by Cai et al., the AHL-lactonase RmML had no reducing effect on PAO1’s extracellular protease activity (37). Different from Aii810, AidH_A147G_ has a strong inhibitory effect on pyocyanin (33).

Until now, few reports about AHL-lactonases have focused on clinical strains. Three pathogenic P. aeruginosa strains were obtained from an infected wound of a burn patient (PA1), an upper-extremity skin injury of a patient with type II diabetes mellitus (PA2), and sputum from a patient with bronchiectasis and infection (PA3). The strain morphologies are shown in Fig. S7. We also tested the production of virulence factors and biofilms by different strains (Fig. S8). The results for biofilms quantified by crystal violet staining showed that the levels of biofilm formation by clinical isolates PA1 and PA3 were higher than that of the wild-type strain PAO1. The concentrations of alginate and LasB secreted by strain PA3 were significantly higher than those secreted by PAO1. The concentrations of pyocyanin secreted by strains PA1 and PA2 were significantly higher than those secreted by PAO1. The concentrations of alginate and LasB secreted by PA2 and the proteolytic activities of PA1 and PA2 were lower than those of PAO1. To evaluate the quorum-quenching effect of AidH_A147G_ on clinical pathogens, we assessed 100 μg/mL of enzymes for the attenuation of the virulence and biofilm formation of P. aeruginosa clinical isolates. Compared with PAO1, the clinical isolates failed to achieve the desired result. For proteolytic activity, the levels of biofilm formation by clinical strains PA1, PA2, and PA3 all decreased by about 10%. For LasB elastase activity, that of clinical strain PA3 revealed inhibition by 80%, exceeding those of clinical strains PA1 and PA2, with reductions of 53.1% and 33.8%, respectively. For alginate (and pyocyanin), the percentages of inhibition were 25%, 30%, and 10% (and 60%, 70%, and 40%) for clinical strains PA1, PA2, and PA3, respectively. The levels of biofilm inhibition of clinical strains PA1, PA2, and PA3 were all about 30% (Fig. 2). All of these results were inferior to those for wild-type strain PAO1.

**FIG 3:**
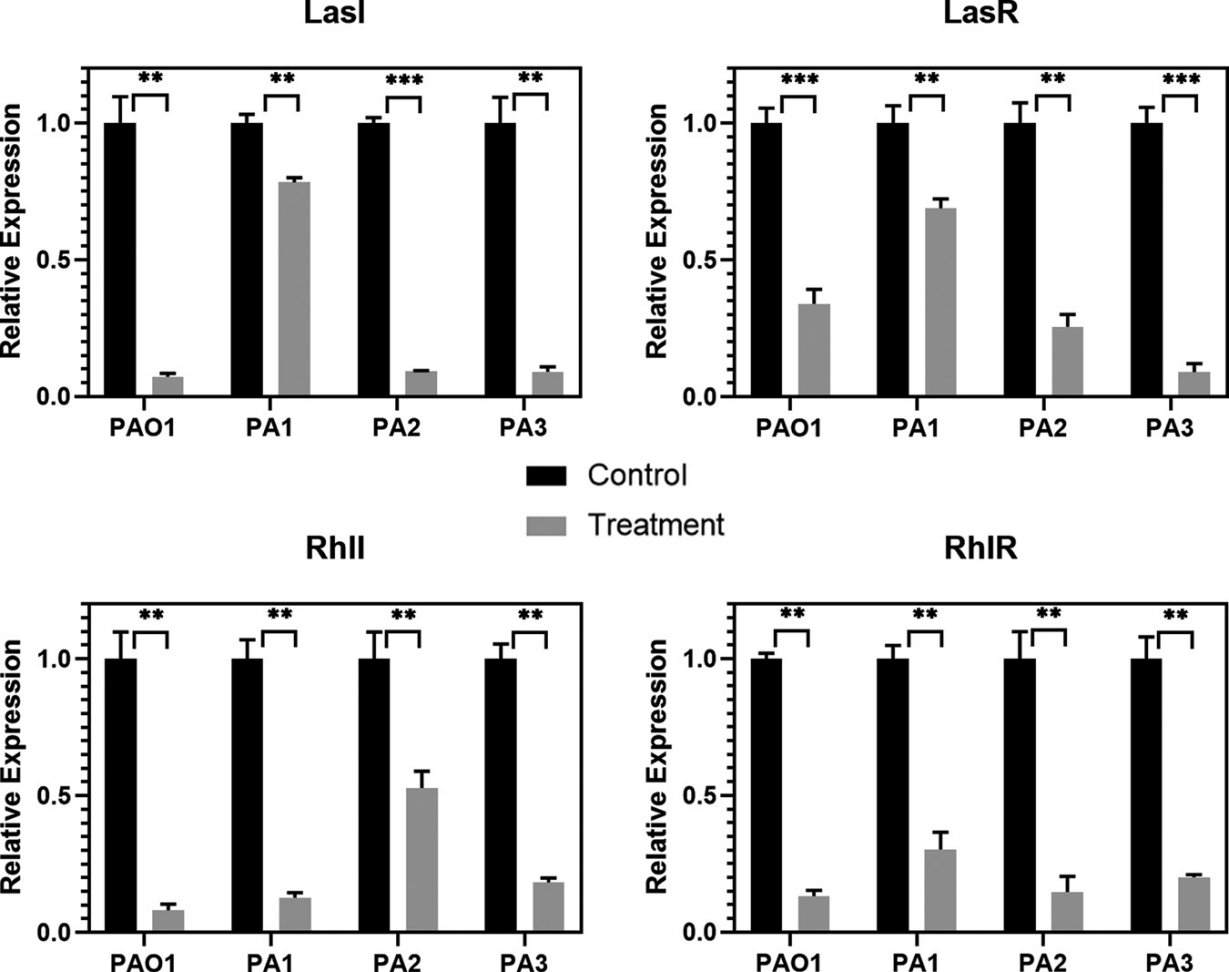
Effect of AidH_A147G_ on the regulation of the expression of QS system genes. PAO1 represents P. aeruginosa wild-type strain PAO1, and PA1, PA2, and PA3 represent the clinical isolates. The ordinate represents relative expression. Each data point represents the mean from three replicates. Error bars in each panel represent SD from triplicates. A *t* test was performed to test differences between groups. *, *P* < 0.05; **, *P* < 0.01; ***, *P* < 0.001.

For the effect of AidH_A147G_ on regulating the expression of QS system genes, qPCR (RT-qPCR) experiments showed that AidH_A147G_ downregulated the relative expression of the Las system and the Rhl system of PAO1 (Fig. 3). This result aligns with the principle of the activity of AHL-lactonases toward the P. aeruginosa QS system. Consistent with the detection of virulence factors, the clinically isolated strains were less effective than PAO1, especially LasI/LasR in clinical strain PA1. Interestingly, clinical strain PA1 displayed multidrug resistance (MDR). It has been confirmed that LasA expression is correlated with antibiotic resistance in P. aeruginosa clinical isolates (38). The LasA gene is regulated mainly by the LasI/LasR system, which may help explain the resistance of clinical strain PA1.

The pathogenicity profile of P. aeruginosa includes a large and variable set of virulence factors and antibiotic resistance genes that allow it to adapt to multiple conditions (39). QS inhibitors may attenuate pathogenicity and drug resistance without affecting growth; thus, choosing a pathway to inhibit biofilms is a win-win approach (40). In contrast, the mutual promotion of the secretion of virulence factors and biofilm formation leads to treatment failure and infection recurrence. The virulence factors associated with the QS system (e.g., esterases, lipases, and elastases) affect extracellular polymeric substance compositions, extracellular matrix properties, and cell motility, thereby affecting P. aeruginosa biofilm formation and structure (41, 42). Proteolytic enzymes affect biofilm formation through rhamnolipid regulation (40). The P. aeruginosa metabolite pyocyanin is associated with biofilm development (43). In addition, biofilms help create robust environmental conditions for the production of virulence factors. Environmental stress could exert substantial effects on QS systems. These factors make the biofilm a potentially effective target for clinical applications.

### Combined enzymes could block the formation of biofilms by P. aeruginosa.

The quantitative detection of virulence factors and biofilms demonstrated that the clinical strains were more difficult to suppress than the PAO1 strain using AidH_A147G_ singly. Moreover, when antimicrobial therapy is delayed, allowing the biofilm to mature, the directly targeted destruction of biofilms is then required. Induced dispersal is one strategy to revert biofilm bacteria to a planktonic state, causing them to lose their resistance to antibiotics (9). Therefore, we propose combining anti-QS and biofilm dispersal to simultaneously prevent biofilm formation and break down established biofilms. PslG, a self-produced glycosyl hydrolase, efficiently breaks up biofilms of P. aeruginosa at nanomolar concentrations and is nontoxic *in vitro* and *in vivo* (23, 26, 27). Thus, PslG was chosen as a tool to induce dispersion in our study.

**FIG 4:**
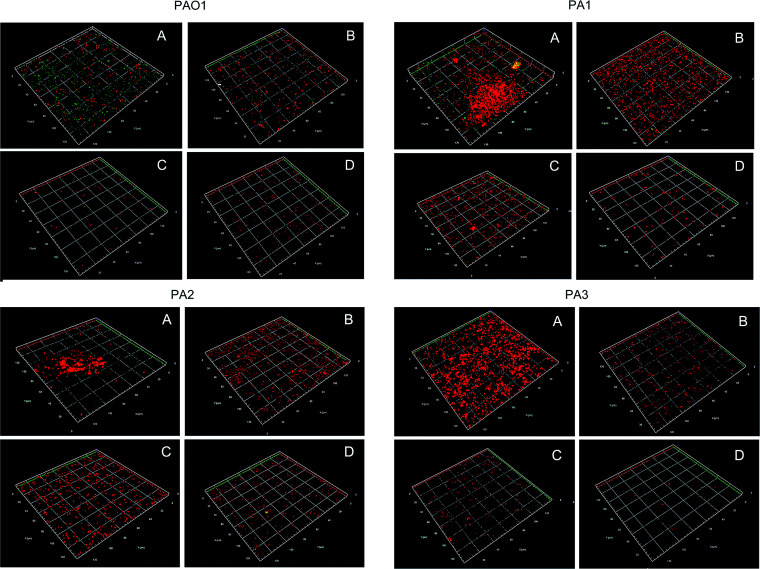
Confocal laser scanning microscopy (CLSM) images of biofilm formation on NEST glass-bottom cell culture dish. Biofilms were incubated for 72 h at 37°C by means of re-adding the enzyme at intervals of 24 h. (A) Control group; (B) cultures with 100 μg/mL AidH_A147G_; (C) cultures with 25 μg/mL PslG; (D) cultures with 25 μg/mL PslG and 100 μg/mL AidH_A147G_. PAO1 represents P. aeruginosa wild-type strain PAO1, and PA1, PA2, and PA3 represent the clinical isolates.

**FIG 5:**
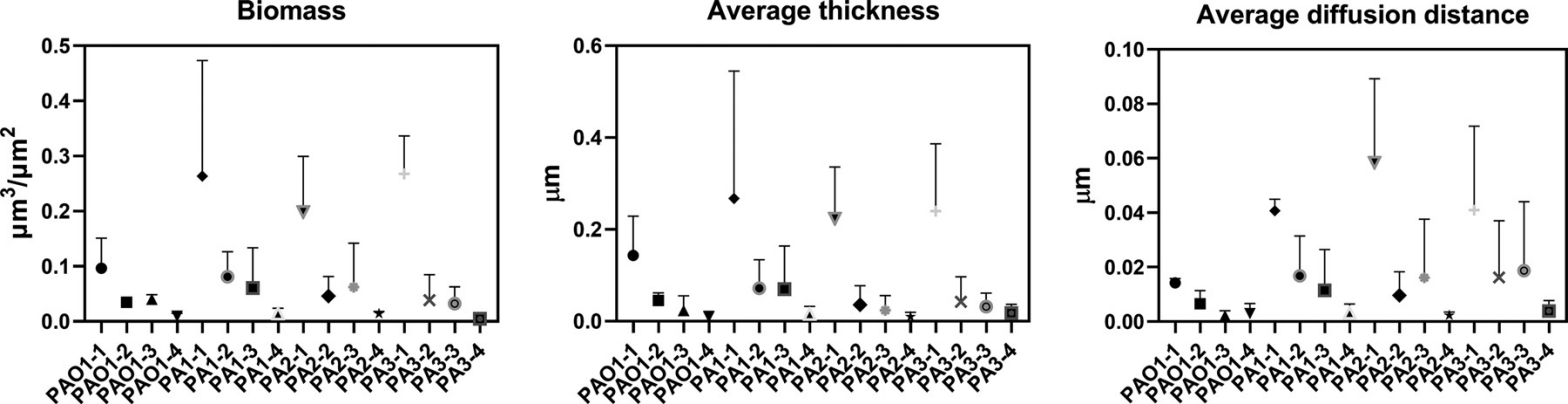
Biofilm biomass, average thickness, and average diffusion distance quantified by COMSTAT software. -1 represents the control group, -2 represents the cultures with 100 μg/mL AidH_A147G_, -3 represents the cultures with 25 μg/mL PslG, and -4 represents the cultures with 25 μg/mL PslG and 100 μg/mL AidH_A147G_. PAO1 represents P. aeruginosa wild-type strain PAO1, and PA1, PA2, and PA3 represent the clinical isolates.

To investigate the inhibition and disruption activities against biofilms, confocal laser scanning microscopy (CLSM) was used to detect biofilms of wild-type strains and clinical pathogenic strains (Fig. 4). In addition, the strategy of anti-QS or induced dispersion was compared with the combination strategy. Using COMSTAT data, we analyzed the following biofilm structural properties: biomass, average thickness, and average diffusion distance (Fig. 5 and Table 2). Without intervention, the biofilm indices of the three clinical isolates were significantly higher than those of wild-type strain PAO1, indicating that the clinical isolates exhibited a stronger capacity for biofilm formation. Comparing different intervention groups, single-enzyme intervention led to an obvious antibiofilm effect on PAO1, and there was no significant difference between AidH_A147G_ and PslG. For clinical strain PA1 and clinical strain PA3, small differences appeared between the PslG group and the AidH_A147G_ group. However, for clinical strain PA2, the anti-QS strategy was more effective. Interestingly, under combined-enzyme intervention for PAO1 and clinical strains, no biofilm was observed on the bottom of NEST glass-bottom cell culture dishes. For the other AHL-lactonases Aii810, AiiA, PvdQ, Gcl, and SsoPox, the biofilm amounts were reduced by about 70%, 80%, 50%, 60%, and 60%, respectively (33, 44–46). Other enzymes dispersed the biofilm by about 70% (23, 47, 48). In addition, two quorum-sensing-quenching enzymes (SsoPox W263I and Gcl) could completely inhibit biofilm formation (46). However, the two enzymes targeted only quorum quenching and did not disrupt established biofilms. The results described above demonstrate that our combined-enzyme approach for inhibiting biofilms is a potential clinical treatment.

**TABLE 2:** Biofilm biomass, surface, and thickness quantified by COMSTAT software^*a*^

Group	Mean biomass (μm^3^/μm^2^) ± SD	Avg thickness (μm) ± SD	Avg diffusion distance (μm) ± SD
PAO1-1	0.097 ± 0.054	0.143 ± 0.085	0.014 ± 0.002
PAO1-2	0.035 ± 0.009	0.045 ± 0.016	0.006 ± 0.005
PAO1-3	0.040 ± 0.008	0.023 ± 0.032	0.002 ± 0.002
PAO1-4	0.009 ± 0.006	0.009 ± 0.012	0.003 ± 0.004
PA1-1	0.264 ± 0.209	0.267 ± 0.278	0.041 ± 0.004
PA1-2	0.081 ± 0.046	0.072 ± 0.061	0.017 ± 0.015
PA-3	0.061 ± 0.073	0.070 ± 0.094	0.011 ± 0.015
PA1-4	0.014 ± 0.009	0.016 ± 0.016	0.003 ± 0.003
PA2-1	0.199 ± 0.100	0.223 ± 0.113	0.058 ± 0.031
PA2-2	0.046 ± 0.035	0.036 ± 0.041	0.010 ± 0.009
PA2-3	0.062 ± 0.079	0.024 ± 0.032	0.016 ± 0.022
PA2-4	0.014 ± 0.004	0.010 ± 0.009	0.002 ± 0.001
PA3-1	0.268 ± 0.069	0.239 ± 0.146	0.041 ± 0.031
PA3-2	0.039 ± 0.046	0.042 ± 0.054	0.016 ± 0.021
PA3-3	0.032 ± 0.030	0.032 ± 0.029	0.019 ± 0.025
PA3-4	0.005 ± 0.001	0.018 ± 0.019	0.004 ± 0.004

a -1 represents the control group, -2 represents the cultures with 100 μg/mL AidH_A147G_, -3 represents the cultures with 25 μg/mL PslG, and -4 represents the cultures with 25 μg/mL PslG and 100 μg/mL AidH_A147G_. PAO1 represents P. aeruginosa wild-type strain PAO1, and PA1, PA2, and PA3 represent the clinical isolates.

### Effect of combined enzymes on the therapeutic efficacy of antibiotics.

**FIG 6:**
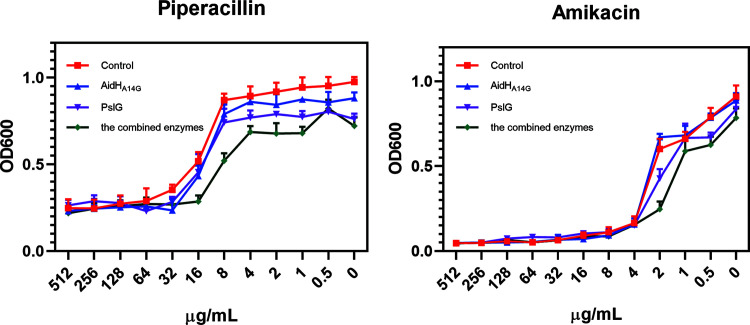
MIC results for wild-type strain PAO1. The antibiotic concentrations ranged from 0.5 to 512 μg/mL. Totals of 100 μg/mL AidH_A147G_, 25 μg/mL PslG, and the mixed solution of both enzymes were added to the three experimental groups. The same volume of sodium phosphate buffer was added to the control group. Error bars in each panel represent SD from triplicates.

Biofilm-targeting enzymes dramatically prevent and break down biofilms, neither of which can inhibit the growth of planktonic bacteria that can lead to severe sepsis in the blood (49). Biofilm-targeting enzymes are thus used as auxiliary therapies in conjunction with antibiotics. To explore whether enzyme therapy enhances the sensitivity of biofilm bacteria to antibiotics, piperacillin, a representative drug of the β-lactams, and amikacin, a representative drug of the aminoglycosides, were chosen to assess the MICs of PAO1. The MIC results revealed that the single-enzyme and combined-enzyme treatments yielded different degrees of antibiotic sensitivity improvements (Fig. 6). The MIC of piperacillin in the control group was 64 μg/mL, and the MICs in the AidH_A147G_ and PslG single-target groups were both 32 μg/mL. For the combination group, the MIC dropped to 16 μg/mL. The MICs of amikacin in both the control group and the AidH_A147G_ and PslG single-target groups were 4 μg/mL, and the MIC for the combined group decreased to 2 μg/mL. Consistent with the biofilm inhibition results, these results showed that the combination strategy led to the most significant increase in antibiotic susceptibility.

The antibiotic susceptibility results for the three P. aeruginosa clinical strains were judged according to 2021 CLSI standards (50), and antimicrobial resistance was defined according to research reported previously by Rossolini et al. (7). Clinical isolates PA1 and PA3 were MDR strains, and PA2 was an aminoglycoside-resistant strain. For the clinical strains, MicroScan MIC panel evaluation based on the microdilution method was used to evaluate the adjuvant effects of the enzymes. This method evaluated 24 drugs commonly used in clinical practice, including several classes of antibiotics (e.g., β-lactams, aminoglycosides, quinolones, and polypeptides) used to treat P. aeruginosa infection (7, 51, 52).

In the cultures for the MicroScan MIC panels, the enzymatic intervention increased the susceptibilities to eight antibiotics, as shown in Table 3. For the aminoglycoside antibiotic tobramycin, the three experimental groups were equally effective in reverting MDR PA1 from a resistant state to an intermediate state. For aminoglycoside-resistant strain PA2, the combined-enzyme intervention performed better than the single-enzyme approach in assisting tobramycin to induce a switch from the resistant to the intermediate state. The combined-enzyme approach changed MDR PA3 from resistant to sensitive, the most striking result. With gentamicin, AidH_A147G_ and combined enzymes helped the PA3 strain change from the intermediate to the sensitive state. For the quinolone antibiotic ciprofloxacin, the interventions with both PslG and combined enzymes led PA1 bacteria to change from the intermediate to the sensitive state.

**TABLE 3:** Effect of combined enzymes on the efficacy of several usual antibiotics^*a*^

Group	Gentamicin		Ciprofloxacin		Tobramycin		Meropenem		Piperacillin-tazobactam		Cefepime		Ceftriaxone MIC (μg/mL)	Ceftazidime	
	MIC (μg/mL)	Resistance state	MIC (μg/mL)	Resistance state	MIC (μg/mL)	Resistance state	MIC (μg/mL)	Resistance state	MIC (μg/mL)	Resistance state	MIC (μg/mL)	Resistance state		MIC (μg/mL)	Resistance state
PA1-1	>8	R	2	I	>8	R	8	I	>64	R	>16	R	>32	>16	R
PA1-2	>8	R	2	I	8	I	4	S	>64	R	>16	R	>32	>16	R
PA1-3	>8	R	≤1	S	8	I	8	I	>64	R	>16	R	>32	>16	R
PA1-4	>8	R	≤1	S	8	I	4	S	>64	R	>16	R	>32	>16	R
PA2-1	≤4	S	≤1	S	>8	R	4	S	>64	R	16	I	>32	16	I
PA2-2	≤4	S	≤1	S	>8	R	4	S	>64	R	≤4	S	>32	16	I
PA2-3	≤4	S	≤1	S	>8	R	4	S	>64	R	16	I	>32	16	I
PA2-4	≤4	S	≤1	S	8	I	4	S	>64	R	≤4	S	>32	16	I
PA3-1	8	I	≤1	S	>8	R	8	I	>64	R	16	I	>32	>16	R
PA3-2	≤4	S	≤1	S	8	I	2	S	>64	R	16	I	>32	4	S
PA3-3	8	I	≤1	S	8	I	8	I	>64	R	8	S	32	4	S
PA3-4	≤4	S	≤1	S	≤4	S	2	S	≤16	S	≤4	S	32	4	S

a R represents drug resistance, I represents intermediate drug resistance, and S represents drug sensitivity. PA1, PA2, and PA3 represent the clinical isolates. -1 represents the control group, -2 represents the cultures with 100 μg/mL AidH_A147G_, -3 represents the cultures with 25 μg/mL PslG, and -4 represents the cultures with 25 μg/mL PslG and 100 μg/mL AidH_A147G_.

Among the β-lactam antibiotics, the combined-enzyme method effectively increased the sensitivity of P. aeruginosa to cephalosporins, carbapenems, and penicillins (Table 3). For cephalosporins, the intervention is most remarkable for PA3. The auxiliary effect can promote the antibacterial activities of cefepime (fourth generation), ceftriaxone (third generation), and ceftazidime (third generation). For cefepime, PA2 changed from an intermediate to a sensitive state under the AidH_A147G_ intervention and the combination intervention. The multidrug-resistant PA1 and PA3 strains changed from an intermediate to a sensitive state after the administration of meropenem in conjunction with AidH_A147G_ or the combined-enzyme method. The combined-enzyme method was more effective than the single-enzyme method when used in conjunction with penicillin antibiotics (e.g., piperacillin and tazobactam). Although not all resistant clinical bacterial strains became sensitive, our combined-enzyme treatment gave the best performance among the enzyme-targeting strategies so far. In fact, bacterial resistance is multifactorial. Besides biofilm resistance, there are intrinsic resistance mechanisms, including low outer membrane permeability, the expression of efflux pumps that expel antibiotics out of cells, and the production of antibiotic-inactivating enzymes (53, 54). Therefore, the exploration of bacterial resistance is complicated and significative.

### Conclusion.

Bacterial biofilms have strong resistance to antibiotics, endangering human health. Biofilm inhibition is a promising research direction for the treatment of biofilm-associated infections. In our research, a combined-enzyme-targeting strategy was first put forward to simultaneously prevent biofilm formation and break down preformed biofilms. The combination could completely disrupt the biofilms of P. aeruginosa wild-type strain PAO1 and multidrug-recalcitrant clinical strains. More importantly, the combination strategy also helped multidrug-resistant clinical strains change from a resistant to an intermediate or sensitive state and potentiated the killing of bacteria by many common antibiotics. These results make it an attractive therapeutic strategy against P. aeruginosa infection.

## MATERIALS AND METHODS

### Chemicals and materials.

AHLs, *p*-nitrophenyl acetate, azocasein, elastin-Congo red, propidium iodide (PI), and fluorescein isothiocyanate (FITC)-concanavalin A (ConA) were purchased from Sigma-Aldrich (St. Louis, MO, USA). The DNA ligation kit, restriction endonuclease, DNA polymerase PrimeScript RT reagent kit, and TB green Premix ExTaq II were purchased from TaKaRa (Dalian, China) and used according to the manufacturer’s recommendations. The E.Z.N.A. plasmid minikit and the E.Z.N.A. gel extraction kit were purchased from Omega (Norcross, GA, USA). The RNeasy MinElute cleanup kit was purchased from Qiagen (Hilden, Germany). All other chemicals and reagents were of analytical grade and purchased from commercial sources unless otherwise stated.

### Bacterial strains and plasmids.

Escherichia coli BL21(DE3) (Tolobio, Shanghai, China) was used as the host for molecular cloning, and E. coli DH5α (Tolobio) was used for plasmid construction. The pET-28a(+) vector (Novagen, Madison, WI, USA) was used to express the target protein. Wild-type strain P. aeruginosa PAO1 was a laboratory-stored strain. P. aeruginosa clinical isolates were kindly provided by the First Affiliated Hospital of Anhui Medical University.

### Rational design.

The crystal structures of the *N*-acylhomoserine lactonase AidH complexed with *N*-butanoyl homoserine were obtained from the RCSB PDB (PDB accession no. 4G8B). Residues 5 Å away from the ligand were selected and analyzed using PyMOL software. 3-Oxo-C_12_-HSL and C_4_-HSL were used as the substrates for screening for mutants with improved performances. The substrate tunnel was predicted by using the CAVER PyMOL 3.0 plug-in.

### Site-directed mutagenesis.

Plasmid pET28a(+)-*aid*H was used as the template. Overlap extension PCR was implemented using the primeSTAR DNA polymerase (TaKaRa). The procedure was as follows: a denaturation step at 94°C for 5 min and 30 repetitions of a cycle including denaturation at 94°C for 30 s, annealing depending on the melting temperature (*T_m_*) value for 30 s, extension at 72°C for 5 s, and a final extension step at 72°C for 10 min. Primers used in this study are listed in Table S2 in the supplemental material.

### Protein expression and purification.

The *aidH* gene (GenBank accession no. GQ849010) and mutants were cloned into pET28a(+) using the BamHI and HindIII restriction sites. The entire *pslG* gene was cloned from the genomic DNA of P. aeruginosa PAO1 and cloned into pET28a(+) at the NdeI and HindIII sites. The resulting expression plasmid was transformed into E. coli BL21(DE3) cells, which were cultured in LB medium to an optical density at 600 nm (OD_600_) of 0.5 to 0.6. Protein expression was induced by isopropyl-β-d-thiogalactopyranoside at a final concentration of 0.1 mM at 30°C for 10 h. The cells were harvested, resuspended in sodium phosphate buffer (50 mM, pH 6.8), and disrupted by sonication. The lysate was obtained by centrifugation and purified using a Ni-nitrilotriacetic acid (NTA) His-Bind column. The purified protein was dialyzed in sodium phosphate buffer (50 mM, pH 6.8) for 3 h. The proteins were then subjected to a final purification step via a size exclusion chromatography column (AidH_A147G_, Superdex 75 Increase 10/300 GL; PslG, Superdex 200 Increase 10/300 GL) using an Äkta purification system. The purification step was repeated until the protein was determined to be >95% pure by the normalization of the peak area using a fast protein liquid chromatography (FPLC) Äkta purifier. Next, the purity of the protein was determined by SDS-PAGE.

### Specific activity and kinetics of AidH.

To determine enzymatic properties, the method of Fan et al. was adopted, with some modifications (34). First, *p*-nitrophenyl acetate (C_2_) was used as a substrate to determine the relative activity. The substrates at a final concentration of 40 μM were dissolved in 50 mM sodium phosphate buffer (pH 7.5) containing methanol (4%). Hydrolysis for all reactions was performed for 4 min. The production of *p*-nitrophenyl acetate was monitored spectrophotometrically at 405 nm using a Multiskan Go microplate reader. The optimum temperature was determined in the range of 20°C to 50°C, and the optimum pH was determined in the range of pH 6 to 9. A sodium dihydrogen phosphate-citric acid buffer was used in the range of pH 5 to 8. Tris-HCl buffer was used in the range of pH 8 to 9. Thermostability was measured by preincubating the enzyme in 50 mM sodium phosphate buffer (pH 6.8) for 0 h, 1 h, 2 h, 5 h, 8 h, 12 h, and 24 h at each temperature (20°C, 30°C, 37°C, 40°C, and 50°C).

To evaluate the degradation rate and kinetics of AidH for the signaling molecules C_4_-HSL and 3-oxo-C_12_-HSL, a colorimetry assay developed previously by Yang et al. (55) was used to quickly detect activity across many samples. A slight modification was made in the final step by adding 70 μL of a 1:1 mixture of ferric chloride (10% in 4 M HCl) and 95% ethanol. The quantities of various lactones were monitored spectrophotometrically at 520 nm using a 96-well plate and a microplate reader. Reaction mixtures contained sodium phosphate buffer (50 mM, pH 6.8), 5% methanol substrate (for the degradation rate, 1 mM; for kinetics, varying concentrations), and protein (varying concentrations). The reaction took place at 37°C for 10 min.

### Effect of AidH_A147G_ on the regulation of the expression of QS system genes of P. aeruginosa.

Cultures of P. aeruginosa grown overnight were diluted to an OD_600_ of 0.1 in LB medium in a 12-well cell culture plate with different concentrations of purified AidH_A147G_. After incubation under static conditions for 24 h at 37°C, the cells were harvested for RNA extraction, which was performed using the RNeasy MinElute cleanup kit. Real-time PCR and qPCR were performed separately using a PrimeScript RT reagent kit. The qPCR primers used in this study are listed in Table S2.

### Effect of AidH_A147G_ on the attenuation of the virulence of P. aeruginosa.

Cultures of P. aeruginosa grown overnight were diluted to an OD_600_ of 0.1 in LB medium in a 12-well cell culture plate with different concentrations of purified AidH_A147G_. After incubation under static conditions for 24 h at 37°C, the supernatants were filtered and then used for virulence assays.

For the biofilm inhibition assay, planktonic cells were removed, and the 12-well cell culture plates were mildly washed with phosphate-buffered saline (PBS) three times. The biofilm was quantified via crystal violet (CV) staining (500 μL; 1%, wt/vol) and then stained with CV after 30 min. For biofilm disassembly, the culture plates were washed three times with PBS and dried for 20 min. Next, the stained culture plates were destained using 95% ethanol. After 10 min, the quantities of biofilms were determined spectrophotometrically at 590 nm using a 96-well plate and a multiscanner.

For other virulence factors, the method described above was used, with some modifications. Proteolytic activity and LasB elastase levels were measured using azocasein and elastin-Congo red as the substrates, according to methods described previously by Ayora and Götz (56) and Ohman et al. (57). Pyocyanin production by PAO1 was measured according to methods described previously by Essar et al. (58), using trichloromethane for extraction and 0.2 mol/L HCl as the chromogenic reagent. Alginate was measured according to methods described previously by Yasuda et al. (59).

### Effect of enzymes on the formation of biofilms by P. aeruginosa.

After diluting the P. aeruginosa culture overnight in LB medium to an OD_600_ of 0.1, 2 mL of the culture was added to NEST glass-bottom cell culture dishes to form biofilms. The cultures were divided into three experimental groups and one control group. For the experimental groups, 100 μg/mL AidH_A147G_, 25 μg/mL PslG, and the mixed enzymes (100 μg/mL AidH_A147G_ and 25 μg/mL PslG) were separately added. For the control group, we added the same volume of inactivated enzyme liquid. Biofilms were incubated for 72 h at 37°C by re-adding the enzyme at 24-h intervals. After incubation, the plates were washed mildly with PBS three times.

For cell staining, the plates were stained with PI for 15 min and then washed three times with PBS. For biofilm staining, the plates were stained with FITC-ConA for 30 min and then washed three times with PBS. Next, 2.5% glutaraldehyde was applied to fix the cells and biofilms. Every plate was conserved using 60% glycerin at 4°C.

Biofilm formation was detected using confocal laser scanning microscopy. The extracellular polysaccharide signal was received under an excitation light wavelength of 488 nm, and the red cell signal was received under an excitation light wavelength of 535 nm. The biofilm biomass and thickness were quantified using COMSTAT software (60).

### Assay of the antibiotic susceptibility of P. aeruginosa after enzyme interventions.

The MIC represents the concentration of antibiotics required to inhibit the growth of a planktonic bacterial population, which can reflect the auxiliary effect of enzymes. For P. aeruginosa wild-type strain PAO1, the MIC was estimated using the microdilution method. In a 96-well microtiter plate, the concentration of the antibiotics piperacillin and amikacin ranged from 0.5 to 512 μg/mL. The experiments were divided similarly into four groups. Separately, 100 μg/mL AidH_A147G_, 25 μg/mL PslG, and the mixed enzymes (100 μg/mL AidH_A147G_ and 25 μg/mL PslG) were added to the three experimental groups. For the control group, we added the same volume of inactivated enzyme liquid. The 96-well plates were incubated for 12 h at 37°C. The antibacterial activities of antibiotics were tested spectrophotometrically at 600 nm using a 96-well plate and a multiscanner.

For the clinical isolates, MicroScan MIC panels (MicroScan WalkAway; Siemens, Germany) were chosen to evaluate drug resistance. The experiments were divided similarly into four groups. Separately, 100 μg/mL AidH_A147G_, 25 μg/mL PslG, and the mixed enzymes (100 μg/mL AidH_A147G_ and 25 μg/mL PslG) were added to the three experimental groups. For the control group, we added the same volume of inactivated enzyme liquid. In addition, the main methods of operation were performed according to the manufacturer’s instructions.

### Data availability.

All data supporting the findings of this study are available from the corresponding author upon request.

## References

[1] Nick JA, Dedrick RM, Gray AL, Vladar EK, Smith BE, Freeman KG, Malcolm KC, Epperson LE, Hasan NA, Hendrix J, Callahan K, Walton K, Vestal B, Wheeler E, Rysavy NM, Poch K, Caceres S, Lovell VK, Hisert KB, de Moura VC, Chatterjee D, De P, Weakly N, Martiniano SL, Lynch DA, Daley CL, Strong M, Jia F, Hatfull GF, Davidson RM. 2022. Host and pathogen response to bacteriophage engineered against *Mycobacterium abscessus* lung infection. Cell 185:1860–1874.e12. 10.1016/j.cell.2022.04.024. 35568033 PMC9840467

[2] Ben Hur D, Kapach G, Wani NA, Kiper E, Ashkenazi M, Smollan G, Keller N, Efrati O, Shai Y. 2022. Antimicrobial peptides against multidrug-resistant *Pseudomonas aeruginosa* biofilm from cystic fibrosis patients. J Med Chem 65:9050–9062. 10.1021/acs.jmedchem.2c00270. 35759644 PMC9289885

[3] Sikdar R, Elias M. 2020. Quorum quenching enzymes and their effects on virulence, biofilm, and microbiomes: a review of recent advances. Expert Rev Anti Infect Ther 18:1221–1233. 10.1080/14787210.2020.1794815. 32749905 PMC7705441

[4] Zhao Y, Mei L, Si Y, Wu J, Shao J, Wang T, Yan G, Wang C, Wu D. 2020. Sodium new houttuyfonate affects transcriptome and virulence factors of *Pseudomonas aeruginosa* controlled by quorum sensing. Front Pharmacol 11:572375. 10.3389/fphar.2020.572375. 33123010 PMC7566558

[5] Horcajada JP, Montero M, Oliver A, Sorlí L, Luque S, Gómez-Zorrilla S, Benito N, Grau S. 2019. Epidemiology and treatment of multidrug-resistant and extensively drug-resistant *Pseudomonas aeruginosa* infections. Clin Microbiol Rev 32:e00031-19. 10.1128/CMR.00031-19. 31462403 PMC6730496

[6] Ciofu O, Tolker-Nielsen T. 2019. Tolerance and resistance of *Pseudomonas aeruginosa* biofilms to antimicrobial agents—how *P. aeruginosa* can escape antibiotics. Front Microbiol 10:913. 10.3389/fmicb.2019.00913. 31130925 PMC6509751

[7] Rossolini GM, Bochenska M, Fumagalli L, Dowzicky M. 2021. Trends of major antimicrobial resistance phenotypes in Enterobacterales and Gram-negative non-fermenters from ATLAS and EARS-net surveillance systems: Italian vs. European and global data, 2008-2018. Diagn Microbiol Infect Dis 101:115512. 10.1016/j.diagmicrobio.2021.115512. 34419741

[8] Davies J. 2007. Microbes have the last word. A drastic re-evaluation of antimicrobial treatment is needed to overcome the threat of antibiotic-resistant bacteria. EMBO Rep 8:616–621. 10.1038/sj.embor.7401022. 17603533 PMC1905906

[9] Lebeaux D, Ghigo JM, Beloin C. 2014. Biofilm-related infections: bridging the gap between clinical management and fundamental aspects of recalcitrance toward antibiotics. Microbiol Mol Biol Rev 78:510–543. 10.1128/MMBR.00013-14. 25184564 PMC4187679

[10] Hall-Stoodley L, Stoodley P, Kathju S, Høiby N, Moser C, Costerton JW, Moter A, Bjarnsholt T. 2012. Towards diagnostic guidelines for biofilm-associated infections. FEMS Immunol Med Microbiol 65:127–145. 10.1111/j.1574-695X.2012.00968.x. 22469292

[11] Soares A, Alexandre K, Etienne M. 2020. Tolerance and persistence of *Pseudomonas aeruginosa* in biofilms exposed to antibiotics: molecular mechanisms, antibiotic strategies and therapeutic perspectives. Front Microbiol 11:2057. 10.3389/fmicb.2020.02057. 32973737 PMC7481396

[12] Behzadi P, Barath Z, Gajdacs M. 2021. It’s not easy being green: a narrative review on the microbiology, virulence and therapeutic prospects of multidrug-resistant *Pseudomonas aeruginosa*. Antibiotics (Basel) 10:42. 10.3390/antibiotics10010042. 33406652 PMC7823828

[13] Sun ZQ, Xi JY, Yang CP, Cong WJ. 2022. Quorum sensing regulation methods and their effects on biofilm in biological waste treatment systems: a review. Front Environ Sci Eng 16:87. 10.1007/s11783-021-1495-2.

[14] Snarr BD, Baker P, Bamford NC, Sato Y, Liu H, Lehoux M, Gravelat FN, Ostapska H, Baistrocchi SR, Cerone RP, Filler EE, Parsek MR, Filler SG, Howell PL, Sheppard DC. 2017. Microbial glycoside hydrolases as antibiofilm agents with cross-kingdom activity. Proc Natl Acad Sci USA 114:7124–7129. 10.1073/pnas.1702798114. 28634301 PMC5502622

[15] Paluch E, Rewak-Soroczyńska J, Jędrusik I, Mazurkiewicz E, Jermakow K. 2020. Prevention of biofilm formation by quorum quenching. Appl Microbiol Biotechnol 104:1871–1881. 10.1007/s00253-020-10349-w. 31927762 PMC7007913

[16] Mukherjee S, Bassler BL. 2019. Bacterial quorum sensing in complex and dynamically changing environments. Nat Rev Microbiol 17:371–382. 10.1038/s41579-019-0186-5. 30944413 PMC6615036

[17] Liu C, Sun D, Liu J, Chen Y, Zhou X, Ru Y, Zhu J, Liu W. 2022. cAMP and c-di-GMP synergistically support biofilm maintenance through the direct interaction of their effectors. Nat Commun 13:1493. 10.1038/s41467-022-29240-5. 35315431 PMC8938473

[18] Miller M, Bassler BL. 2001. Quorum sensing in bacteria. Annu Rev Microbiol 55:165–199. 10.1146/annurev.micro.55.1.165. 11544353

[19] Boursier ME, Moore JD, Heitman KM, Shepardson-Fungairino SP, Combs JB, Koenig LC, Shin D, Brown EC, Nagarajan R, Blackwell HE. 2018. Structure-function analyses of the N-butanoyl l-homoserine lactone quorum-sensing signal define features critical to activity in RhlR. ACS Chem Biol 13:2655–2662. 10.1021/acschembio.8b00577. 30114353 PMC6200399

[20] Billot R, Plener L, Jacquet P, Elias M, Chabrière E, Daudé D. 2020. Engineering acyl-homoserine lactone-interfering enzymes toward bacterial control. J Biol Chem 295:12993–13007. 10.1074/jbc.REV120.013531. 32690609 PMC7489903

[21] Flemming HC, Wingender J. 2010. The biofilm matrix. Nat Rev Microbiol 8:623–633. 10.1038/nrmicro2415. 20676145

[22] Ma L, Wang J, Wang S, Anderson EM, Lam JS, Parsek MR, Wozniak DJ. 2012. Synthesis of multiple *Pseudomonas aeruginosa* biofilm matrix exopolysaccharides is post-transcriptionally regulated. Environ Microbiol 14:1995–2005. 10.1111/j.1462-2920.2012.02753.x. 22513190 PMC4446059

[23] Yu S, Su T, Wu H, Liu S, Wang D, Zhao T, Jin Z, Du W, Zhu MJ, Chua SL, Yang L, Zhu D, Gu L, Ma LZ. 2015. PslG, a self-produced glycosyl hydrolase, triggers biofilm disassembly by disrupting exopolysaccharide matrix. Cell Res 25:1352–1367. 10.1038/cr.2015.129. 26611635 PMC4670989

[24] Mei G-Y, Yan X-X, Turak A, Luo Z-Q, Zhang L-Q. 2010. AidH, an alpha/beta-hydrolase fold family member from an *Ochrobactrum* sp. strain, is a novel *N*-acylhomoserine lactonase. Appl Environ Microbiol 76:4933–4942. 10.1128/AEM.00477-10. 20525860 PMC2916461

[25] Gao A, Mei GY, Liu S, Wang P, Tang Q, Liu YP, Wen H, An XM, Zhang LQ, Yan XX, Liang DC. 2013. High-resolution structures of AidH complexes provide insights into a novel catalytic mechanism for N-acyl homoserine lactonase. Acta Crystallogr D Biol Crystallogr 69:82–91. 10.1107/S0907444912042369. 23275166 PMC3532132

[26] Pestrak MJ, Baker P, Dellos-Nolan S, Hill PJ, Passos da Silva D, Silver H, Lacdao I, Raju D, Parsek MR, Wozniak DJ, Howell PL. 2019. Treatment with the *Pseudomonas aeruginosa* glycoside hydrolase PslG combats wound infection by improving antibiotic efficacy and host innate immune activity. Antimicrob Agents Chemother 63:e00234-19. 10.1128/AAC.00234-19. 30988141 PMC6535529

[27] Baker P, Whitfield GB, Hill PJ, Little DJ, Pestrak MJ, Robinson H, Wozniak DJ, Howell PL. 2015. Characterization of the *Pseudomonas aeruginosa* glycoside hydrolase PslG reveals that its levels are critical for Psl polysaccharide biosynthesis and biofilm formation. J Biol Chem 290:28374–28387. 10.1074/jbc.M115.674929. 26424791 PMC4653695

[28] Kyeong HH, Kim JH, Kim HS. 2015. Design of N-acyl homoserine lactonase with high substrate specificity by a rational approach. Appl Microbiol Biotechnol 99:4735–4742. 10.1007/s00253-014-6304-4. 25547834

[29] Hiblot J, Gotthard G, Elias M, Chabriere E. 2013. Differential active site loop conformations mediate promiscuous activities in the lactonase SsoPox. PLoS One 8:e75272. 10.1371/journal.pone.0075272. 24086491 PMC3781021

[30] Li XC, Wang C, Mulchandani A, Ge X. 2016. Engineering soluble human paraoxonase 2 for quorum quenching. ACS Chem Biol 11:3122–3131. 10.1021/acschembio.6b00527. 27623343

[31] Koch G, Nadal-Jimenez P, Reis CR, Muntendam R, Bokhove M, Melillo E, Dijkstra BW, Cool RH, Quax WJ. 2014. Reducing virulence of the human pathogen *Burkholderia* by altering the substrate specificity of the quorum-quenching acylase PvdQ. Proc Natl Acad Sci USA 111:1568–1573. 10.1073/pnas.1311263111. 24474783 PMC3910591

[32] Chow JY, Xue B, Lee KH, Tung A, Wu L, Robinson RC, Yew WS. 2010. Directed evolution of a thermostable quorum-quenching lactonase from the amidohydrolase superfamily. J Biol Chem 285:40911–40920. 10.1074/jbc.M110.177139. 20980257 PMC3003391

[33] Fan X, Liang M, Wang L, Chen R, Li H, Liu X. 2017. Aii810, a novel cold-adapted *N*-acylhomoserine lactonase discovered in a metagenome, can strongly attenuate *Pseudomonas aeruginosa* virulence factors and biofilm formation. Front Microbiol 8:1950. 10.3389/fmicb.2017.01950. 29067011 PMC5641347

[34] Dong W, Zhu J, Guo X, Kong D, Zhang Q, Zhou Y, Liu X, Zhao S, Ruan Z. 2018. Characterization of AiiK, an AHL lactonase, from *Kurthia huakui* LAM0618^T^ and its application in quorum quenching on *Pseudomonas aeruginosa* PAO1. Sci Rep 8:6013. 10.1038/s41598-018-24507-8. 29662232 PMC5902575

[35] Fan X, Liu X, Liu Y. 2012. The cloning and characterization of one novel metagenome-derived thermostable esterase acting on N-acylhomoserine lactones. J Mol Catal B Enzym 83:29–37. 10.1016/j.molcatb.2012.07.006.

[36] Morohoshi T, Kamimura Y, Someya N. 2020. Identification and characterization of quorum-quenching activity of N-acylhomoserine lactonase from coagulase-negative staphylococci. Antibiotics (Basel) 9:483. 10.3390/antibiotics9080483. 32764492 PMC7459623

[37] Cai X, Yu M, Shan H, Tian X, Zheng Y, Xue C, Zhang X-H. 2018. Characterization of a novel N-acylhomoserine lactonase RmmL from *Ruegeria mobilis* YJ3. Mar Drugs 16:370. 10.3390/md16100370. 30297643 PMC6213412

[38] Dehbashi S, Pourmand MR, Alikhani MY, Asl SS, Arabestani M. 2020. Coordination of *las* regulated virulence factors with multidrug-resistant and extensively drug-resistant in superbug strains of *P. aeruginosa*. Mol Biol Rep 47:4131–4143. 10.1007/s11033-020-05559-4. 32474845

[39] Duplantier M, Lohou E, Sonnet P. 2021. Quorum sensing inhibitors to quench P. aeruginosa pathogenicity. Pharmaceuticals (Basel) 14:1262. 10.3390/ph14121262. 34959667 PMC8707152

[40] Jurado-Martín I, Sainz-Mejías M, McClean S. 2021. *Pseudomonas aeruginosa*: an audacious pathogen with an adaptable arsenal of virulence factors. Int J Mol Sci 22:3128. 10.3390/ijms22063128. 33803907 PMC8003266

[41] Tielen P, Kuhn H, Rosenau F, Jaeger K-E, Flemming H-C, Wingender J. 2013. Interaction between extracellular lipase LipA and the polysaccharide alginate of *Pseudomonas aeruginosa*. BMC Microbiol 13:159. 10.1186/1471-2180-13-159. 23848942 PMC3733896

[42] Tielen P, Rosenau F, Wilhelm S, Jaeger K-E, Flemming H-C, Wingender J. 2010. Extracellular enzymes affect biofilm formation of mucoid *Pseudomonas aeruginosa*. Microbiology (Reading) 156:2239–2252. 10.1099/mic.0.037036-0. 20360178

[43] VanDrisse CM, Lipsh-Sokolik R, Khersonsky O, Fleishman SJ, Newman DK. 2021. Computationally designed pyocyanin demethylase acts synergistically with tobramycin to kill recalcitrant *Pseudomonas aeruginosa* biofilms. Proc Natl Acad Sci USA 118:e2022012118. 10.1073/pnas.2022012118. 33723058 PMC8000102

[44] Wang TN, Guan QT, Pain A, Kaksonen AH, Hong PY. 2019. Discovering, characterizing, and applying acyl homoserine lactone-quenching enzymes to mitigate microbe-associated problems under saline conditions. Front Microbiol 10:823. 10.3389/fmicb.2019.00823. 31057524 PMC6479171

[45] Vogel J, Wakker-Havinga M, Setroikromo R, Quax WJ. 2020. Immobilized acylase PvdQ reduces *Pseudomonas aeruginosa* biofilm formation on PDMS silicone. Front Chem 8:54. 10.3389/fchem.2020.00054. 32117880 PMC7012999

[46] Rémy B, Plener L, Decloquement P, Armstrong N, Elias M, Daudé D, Chabrière É. 2020. Lactonase specificity is key to quorum quenching in *Pseudomonas aeruginosa*. Front Microbiol 11:762. 10.3389/fmicb.2020.00762. 32390993 PMC7193897

[47] Mahajan S, Sunsunwal S, Gautam V, Singh M, Ramya TNC. 2021. Biofilm inhibitory effect of alginate lyases on mucoid P. aeruginosa from a cystic fibrosis patient. Biochem Biophys Rep 26:101028. 10.1016/j.bbrep.2021.101028. 34095554 PMC8165544

[48] Tavafi H, Ali AA, Ghadam P, Gharavi S. 2018. Screening, cloning and expression of a novel alginate lyase gene from *P. aeruginosa* TAG 48 and its antibiofilm effects on *P. aeruginosa* biofilm. Microb Pathog 124:356–364. 10.1016/j.micpath.2018.08.018. 30118807

[49] Fleming D, Rumbaugh K. 2018. The consequences of biofilm dispersal on the host. Sci Rep 8:10738. 10.1038/s41598-018-29121-2. 30013112 PMC6048044

[50] CLSI. 2021. Performance standards for antimicrobial susceptibility testing, M100, 31st ed. Clinical and Laboratory Standards Institute, Wayne, PA. 10.1128/JCM.00213-21 PMC8601225 34550809

[51] Jangra V, Sharma N, Chhillar AK. 2022. Therapeutic approaches for combating *Pseudomonas aeruginosa* infections. Microbes Infect 24:104950. 10.1016/j.micinf.2022.104950. 35139390

[52] Yang K, Xiao T, Shi Q, Zhu Y, Ye J, Zhou Y, Xiao Y. 2021. Socioeconomic burden of bloodstream infections caused by carbapenem-resistant and carbapenem-susceptible *Pseudomonas aeruginosa* in China. J Glob Antimicrob Resist 26:101–107. 10.1016/j.jgar.2021.03.032. 34023532

[53] Uruén C, Chopo-Escuin G, Tommassen J, Mainar-Jaime RC, Arenas J. 2020. Biofilms as promoters of bacterial antibiotic resistance and tolerance. Antibiotics (Basel) 10:3. 10.3390/antibiotics10010003. 33374551 PMC7822488

[54] Pang Z, Raudonis R, Glick BR, Lin TJ, Cheng Z. 2019. Antibiotic resistance in *Pseudomonas aeruginosa*: mechanisms and alternative therapeutic strategies. Biotechnol Adv 37:177–192. 10.1016/j.biotechadv.2018.11.013. 30500353

[55] Yang Y-H, Lee T-H, Kim JH, Kim EJ, Joo H-S, Lee C-S, Kim B-G. 2006. High-throughput detection method of quorum-sensing molecules by colorimetry and its applications. Anal Biochem 356:297–299. 10.1016/j.ab.2006.05.030. 16857158

[56] Ayora S, Götz F. 1994. Genetic and biochemical properties of an extracellular neutral metalloprotease from *Staphylococcus hyicus* subsp. *hyicus*. Mol Gen Genet 242:421–430. 10.1007/BF00281792. 8121397

[57] Ohman DE, Cryz SJ, Iglewski BH. 1980. Isolation and characterization of *Pseudomonas aeruginosa* PAO mutant that produces altered elastase. J Bacteriol 142:836–842. 10.1128/jb.142.3.836-842.1980. 6769912 PMC294108

[58] Essar DW, Eberly L, Hadero A, Crawford IP. 1990. Identification and characterization of genes for a second anthranilate synthase in *Pseudomonas aeruginosa*: interchangeability of the two anthranilate synthases and evolutionary implications. J Bacteriol 172:884–900. 10.1128/jb.172.2.884-900.1990. 2153661 PMC208517

[59] Yasuda H, Ajiki Y, Koga T, Kawada H, Yokota T. 1993. Interaction between biofilms formed by *Pseudomonas aeruginosa* and clarithromycin. Antimicrob Agents Chemother 37:1749–1755. 10.1128/AAC.37.9.1749. 8239580 PMC188065

[60] Heydorn A, Nielsen AT, Hentzer M, Sternberg C, Givskov M, Ersbøll BK, Molin S. 2000. Quantification of biofilm structures by the novel computer program COMSTAT. Microbiology (Reading) 146:2395–2407. 10.1099/00221287-146-10-2395. 11021916

