# Supplementary material for: Targeting Multidrug-Recalcitrant Pseudomonas aeruginosa Biofilms: Combined-Enzyme Treatment Enhances Antibiotic Efficacy
Source: Antimicrob Agents Chemother. 2023 Jan 5;67(1):e01358-22. doi: 10.1128/aac.01358-22 (PMC9872604; doi:10.1128/aac.01358-22)
Supplement: Supplemental file 1 — Supplemental material. Download aac.01358-22-s0001.pdf, PDF file, 0.3 MB [file aac.01358-22-s0001.pdf]

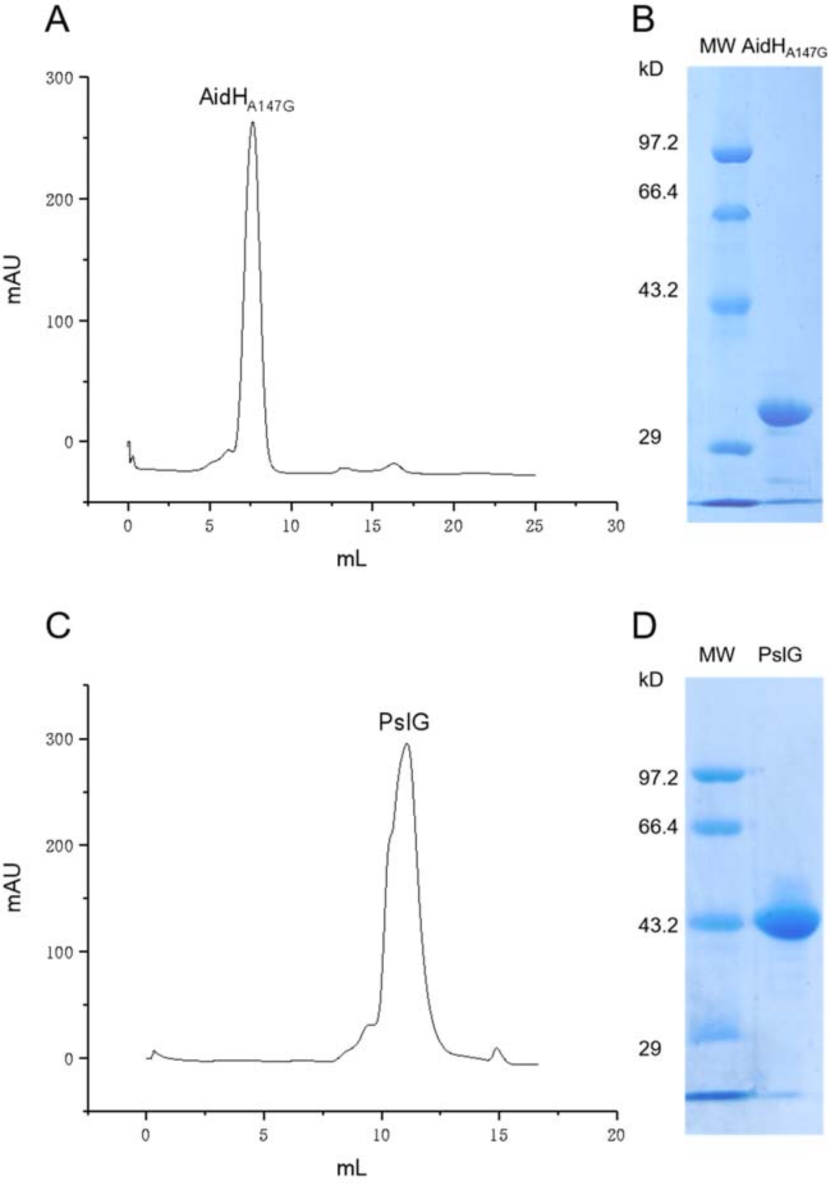

Figure S1. Purification of $AidH_{A147G}$ and PslG. (A) Gel chromatography of $AidH_{A147G}$ using a FPLC-AKTA system, (B) Gel electrophoresis of purified protein $AidH_{A147G}$, (C) Gel chromatography of PslG using a FPLC-AKTA system, (D) Gel electrophoresis of purified protein PslG.

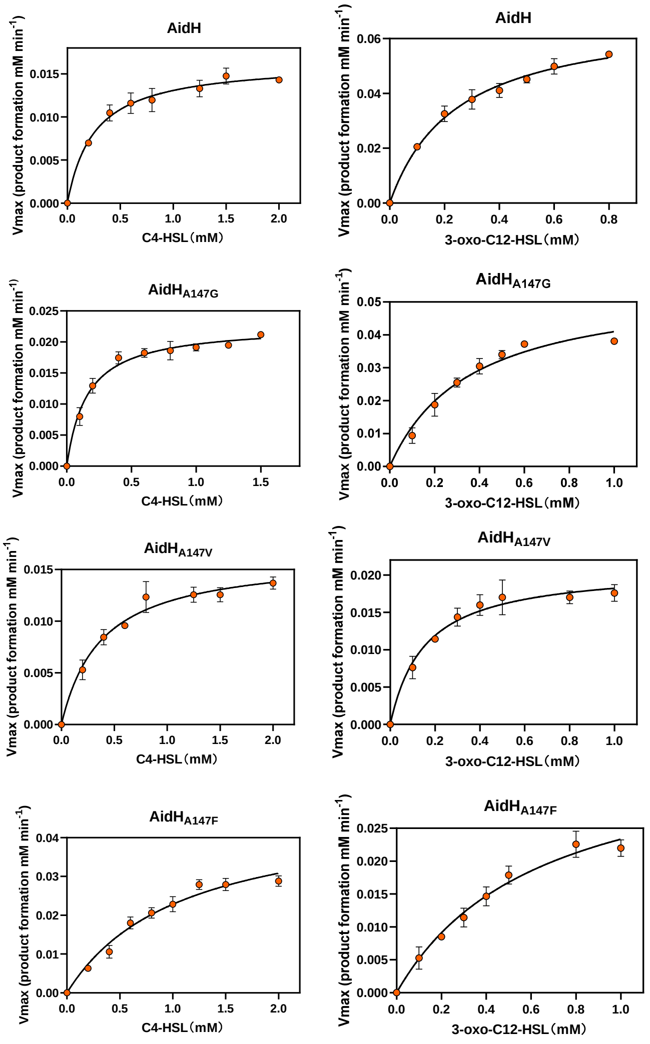

Figure S2. Michaelis-Menten kinetics of AidH and mutants (4.175 nM of AidH for C4-HSL reaction, 16.7 nM of AidH for 3-oxo-C12-HSL reaction, 4.175 nM of AidH$_{A147G}$ for C4-HSL reaction, 8.35 nM of AidH$_{A147G}$ for 3-oxo-C12-HSL reaction, 16.7nM of AidH$_{A147V}$ for C4-HSL reaction,16.7 nM of AidH$_{A147V}$ for 3-oxo-C12-HSL reaction, 33.4 nM of AidH$_{A147F}$ for C4-HSL reaction,42 nM of AidH$_{A147F}$ for 3-oxo-C12-HSL reaction) The abscissa represents substrate concentration (mM), the ordinate represents product formation (mM min^{-1}). Each data point represents the mean of three replicates. Error bars in each panel represent SD of triplicates.

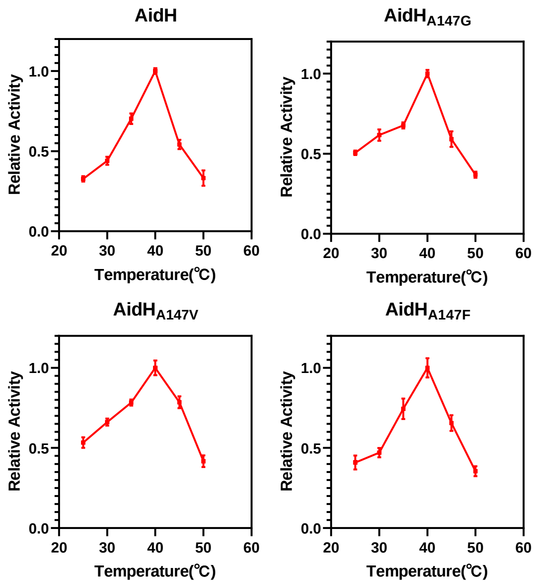

Figure S3. Optimum temperature of AidH and the mutants. The abscissa represents temperature (°C), the ordinate represents relative expression. Each data point represents the mean of three replicates. Error bars in each panel represent SD of triplicates.

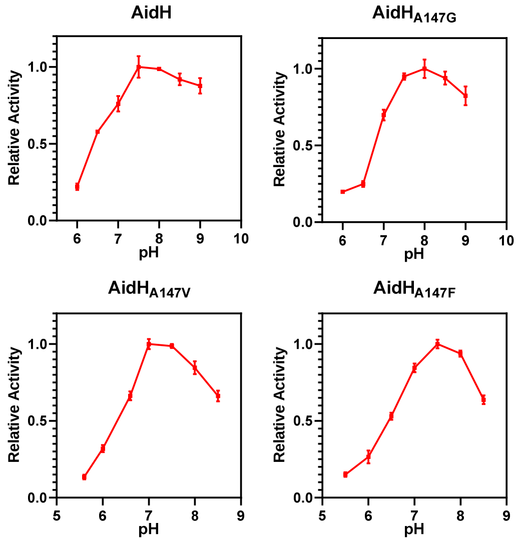

Figure S4. Optimum pH of AidH and the mutants. The abscissa represents different pH, the ordinate represents relative expression. Each data point represents the mean of three replicates. Error bars in each panel represent SD of triplicates.

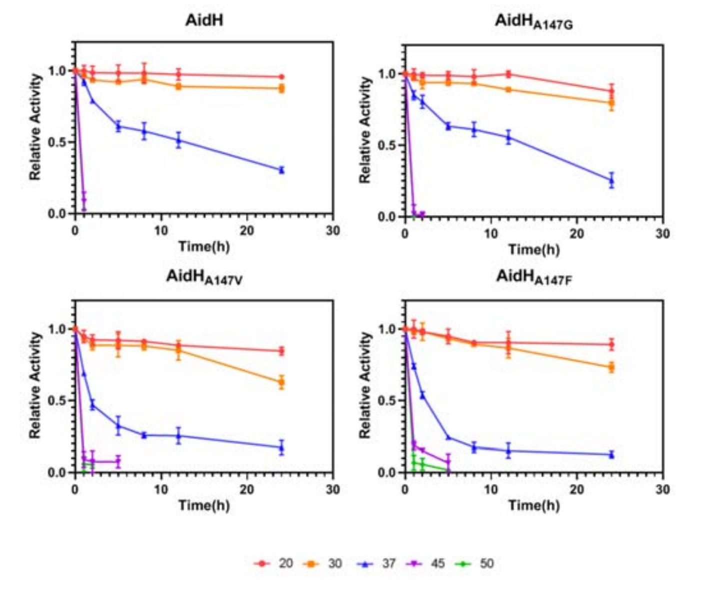

Figure S5. Thermostability of AidH and the mutants. The abscissa represents treatment incubation time, the ordinate represents relative expression. Each data point represents the mean of three replicates. Error bars in each panel represent SD of triplicates.

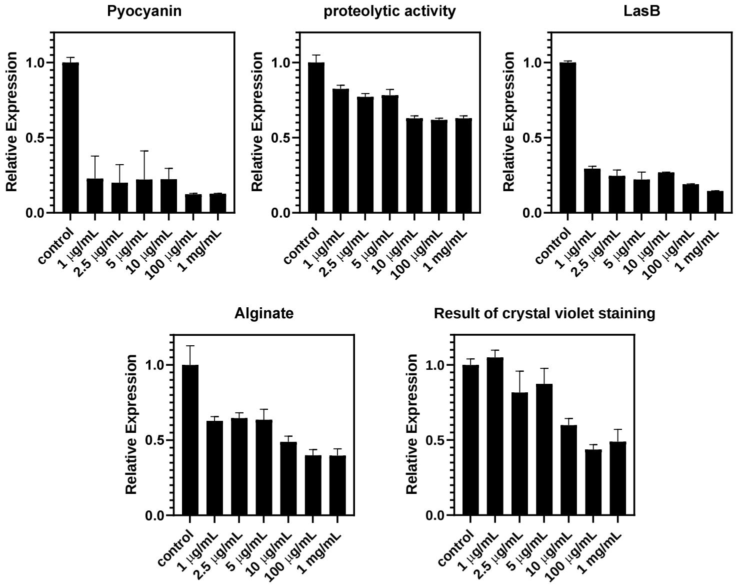

Figure S6. Effect of $AidH_{A147G}$ on the attenuation of virulence in *P. aeruginosa* PAO1. The abscissa represents exertion of different amounts of enzymes, the ordinate represents relative expression. Each data point represents the mean of three replicates. Error bars in each panel represent SD of triplicates.

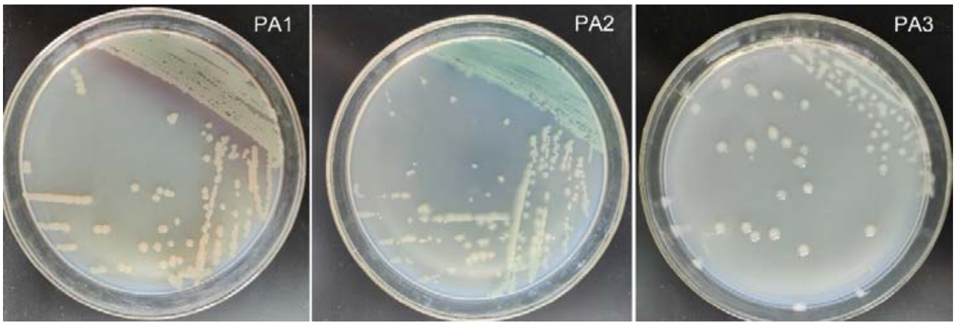

Figure S7. Colony morphology of the strains isolate by clinical cultured on LB agar plates.

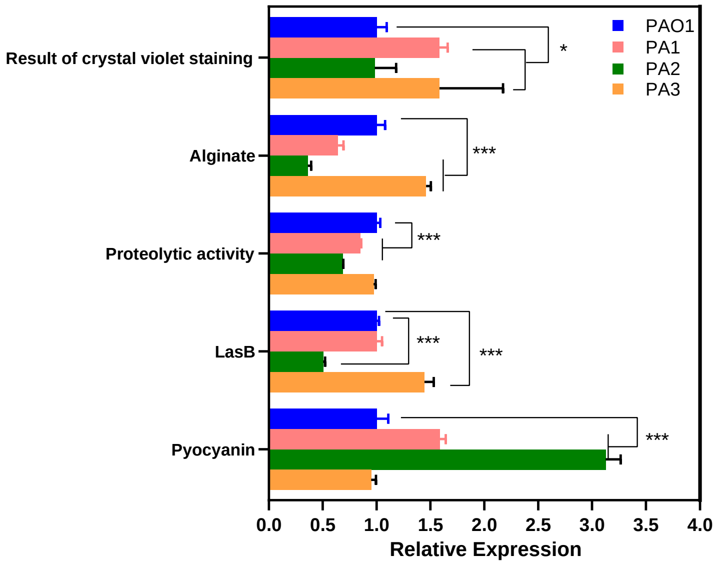

Figure S8. The production of virulence factors and biofilm in different strains. The results of biofilm quantified with crystal violet staining showed that clinical isolates PA1 and PA3 were higher than the wild-type strain PAO1. The content of alginate and LasB secreted by strain PA3 was significantly higher than PAO1. The content of pyocyanin secreted by strain PA1 and PA2 was significantly higher than PAO1. For alginate and LasB secreted by PA2 and proteolytic activity in PA1 and PA2 were lower than PAO1.

Table S1. Degradation rate by AidH and its mutants.

concentration	mutant type	degradation rate of C4-HSL	degradation rate of 3OC12-HSL
1.25 μg/mL	AidH	66.75%±4.54%	12.82%±1.75%
	$AidH_{A147G}$	81.36%±4%	40.21%±2.02%
	$AidH_{A147F}$	10.91%±6.34%	1.16%±2.74%
	$AidH_{A147V}$	40.72%±6.46%	16.32%±3.64%
25 μg/mL	$AidH_{A147V+N33G}$	0.00%	4.44%±1%
	$AidH_{A147V+N33W}$	3.02%±3.78%	4.13%±1.3%

$AidH_{A147V+N33C}$	14.61%±2.14%	6.03%±5.8%
$AidH_{A147V+T164F}$	21.40%±4.54%	10.79%±2.06%
$AidH_{A147V+F221V}$	54.25%±6.24%	63.17%±1.1%

Table S2. Primers used in this study.

Primer Name	Primer Sequence F'	Primer Sequence R'
AidH-F147G	GATATGGCGCTGGGYGGCCAGGA AATTTTTAGC	GCTAAAAATTTCCTGGCCRCCCA GCGCCATATC
AidH-F147V	GATATGGCGCTGGTYGGCCAGGA AATTTTTAGC	GCTAAAAATTTCCTGGCCRACCA GCGCCATATC
AidH-A147F	GATATGGCGCTGTTTGGCCAGGAA ATTTTTAGC	GCTAAAAATTTCCTGGCCAAACA GCGCCATATC

AidH-N33G	GATTCATGGCGGYAGCAGCAGCG GCGCGATTTTTGCG	CGCAAAAATCGCGCCGCTGCTGC TRCCGCCATGAATC
AidH-N33W	GATTCATGGCTGGAGCAGCAGCG GCGCGATTTTTGCG	CGCAAAAATCGCGCCGCTGCTGC TCCAGCCATGAATC
AidH-N33C	GATTCATGGCTGYAGCAGCAGCGG CGCGATTTTTGCG	CGCAAAAATCGCGCCGCTGCTGC TRCAGCCATGAATC
AidH-T164F	CTATGCGCGCAGCTTYTGCGGCGA ACC	GGTTCGCCGCARAAGCTGCGCGC ATAG
AidH-F221V	GATGAACCGGTYGTGGAACTGGA TTTTG	CAAAATCCAGTTCCACRACCGGT TCATC
LasI	CTACAGCCTGCAGAACGACA	ATCTGGGTCTTGGCATTGAG
LasR	ACGCTCAAGTGGAAAATTGG	GTAGATGGACGGTTCCCAGA
RhlI	TCTGGTCCAGCCTGCAATG	TCAGCTTCTGGGTCAGCAACT
RhlR	ATGATGGCGATTTCCCCGGAAC	CATCCGATGCTGATGTCCAACC
PslG	GGGCATATGGAGATCCAGGTACTG AAG	GGGAAGCTTTCACTCCCAGACCA GCA
